# Indole-3-Propionic Acid, a Tryptophan-Derived Bacterial Metabolite, Reduces Weight Gain in Rats

**DOI:** 10.3390/nu11030591

**Published:** 2019-03-11

**Authors:** Piotr Konopelski, Marek Konop, Marta Gawrys-Kopczynska, Piotr Podsadni, Agnieszka Szczepanska, Marcin Ufnal

**Affiliations:** 1 Department of Experimental Physiology and Pathophysiology, Laboratory of Centre for Preclinical Research, Medical University of Warsaw, 02-106 Warsaw, Poland; piotr.konopelski@wum.edu.pl (P.K.); marek.konop@wum.edu.pl (M.K.); marta.gawrys@wum.edu.pl (M.G.-K.); 2 Department of Drug Technology and Pharmaceutical Biotechnology, Medical University of Warsaw, 02-106 Warsaw, Poland; piotr.podsadni@wum.edu.pl (P.P.); agnieszka.szczepanska@wum.edu.pl (A.S.)

**Keywords:** indoles, metabolism, microbiota, tryptophan, weight gain

## Abstract

Recent evidence suggests that tryptophan, an essential amino acid, may exert biological effects by means of tryptophan-derived gut bacteria products. We evaluated the potential contribution of tryptophan-derived bacterial metabolites to body weight gain. The study comprised three experimental series performed on separate groups of male, Sprague-Dawley rats: (i) rats on standard laboratory diet treated with water solution of neomycin, an antibiotic, or tap water (controls-1); (ii) rats on standard diet (controls-2) or tryptophan-high (TH) or tryptophan-free (TF) diet; and (iii) rats treated with indole-3-propionic acid (I3P), a bacterial metabolite of tryptophan, or a vehicle (controls-3). (i) Rats treated with neomycin showed a significantly higher weight gain but lower stool and blood concentration of I3P than controls-1. (ii) The TH group showed significantly smaller increases in body weight but higher stool and plasma concentration of I3P than controls-2. In contrast, the TF group showed a decrease in body weight, decreased total serum protein and a significant increase in urine output. (iii) Rats treated with I3P showed significantly smaller weight gain than controls-3. Our study suggests that I3P, a gut bacteria metabolite of tryptophan, contributes to changes in body weight gain produced by antibiotics and tryptophan-rich diet.

## 1. Introduction

The regulation of appetite and energy homeostasis is a complex set of mechanisms involving numerous biological mediators [1,2,3].

Tryptophan is an essential amino acid, and impairment of its metabolism can lead to serious diseases including pellagra [4], phenylketonuria [5] and Hartnup disease [6]. It has been reported that tryptophan depletion is associated with reduction in body weight, with [7] or without [8,9] concomitant reduction in food intake in rats. Interestingly, a decrease in weight gain and food intake in rats was also found after the introduction of tryptophan-rich diet for two weeks [10]. A similar effect was observed in mice treated with tryptophan by intragastric tube [11] and in rats treated intraperitoneally with tryptophan [12,13]. The mechanism of a such bidirectional effect of tryptophan on body weight gain has not been elucidated.

Recent evidence suggests that tryptophan may exert biological effects not only by endogenously synthetized mediators such as kynurenine and serotonin, but also by tryptophan-derived gut bacteria products, i.e., indoles. The latter include indole-3-acetic acid (I3A), indole-3-propionic acid (I3P), indole-3-lactic acid, (I3L), indole-3-carboxylic acid (I3C), indole and its liver metabolite indoxyl sulfate (IS) [14,15,16,17,18,19]. Research shows the involvement of gut bacteria, and possibly their metabolites, in energy homeostasis. For example, antibiotic treatment in rats promotes increase in body mass index [20]. Furthermore, several antibiotics have been used for decades to increase food intake and weight gain in poultry/broiler chicks [21]. Finally, early-life antibiotic treatment in humans has been associated with increased risk of developing underweight or obesity in the future [22].

The aim of this study was to evaluate the potential link between tryptophan-derived bacterial metabolites and body weight gain in rats.

## 2. Materials and Methods

### 2.1. Animals

The experiments were performed according to Directive 2010/63 EU on the protection of animals used for scientific purposes and approved by the Local Bioethical Committee (no: WAW2/128/2018). Rats were housed in groups (3–4) in polypropylene cages with environmental enrichment, 12 h light/12 h dark cycle, temperature 22–23 °C, humidity 45–55%.

Measurements were performed on male (*n* = 58) Sprague-Dawley rats, fed laboratory diet ad libitum.

### 2.2. The Effect of Antibiotic Treatment on Body Weight Gain and Tryptophan-Derived Bacterial Metabolites Level

12-week-old rats received water solution of neomycin (1 g/1 L, Polfa Tarchomin, Warsaw, Poland) [*n* = 8] or water [n=8] for two weeks. Rats were fed standard laboratory diet (Labofeed B, Morawski, Poland) ad libitum. Body mass, food and water intake, and urine output were measured for two days before and for the last two days of the treatment in metabolic cages. Data from the second day of metabolic measurements were analyzed. Next, rats were anesthetized with intraperitoneal injection of urethane (1.5 g/kg b.w. Sigma-Aldrich, St. Luis, MO, USA) and portal blood and systemic blood from the right ventricle of the heart were collected. Stools from the colon were collected as described below.

### 2.3. The Effect of Tryptophan-Free and Tryptophan-Rich Diet on Body Weight Gain and Bacterial Metabolites Level

12-week-old rats received tryptophan-free (TF group, Tryptophan free crystalline AA diet, Ssniff-Spezialdiäten GmbH, Soest Germany) [*n* = 8], tryptophan-high (TH group, 8.5 g/kg, SM R/M +6 g/kg Tryptophan, Ssniff-Spezialdiäten GmbH, Germany) [*n* = 8] or standard control diet (TC group, TRP 2 g/kg, Labofeed B, Morawski, Poland) [*n* = 8] and water ad libitum for two weeks (Table 1). Body mass, food and water intake, and urine output were measured for two days before and for the last two days of the treatment in metabolic cages. Data from the second day of metabolic measurements were analyzed. After the last measurements, rats were anesthetized with urethane (1.5 g/kg b.w. Sigma-Aldrich); portal blood and systemic blood from the right ventricle of the heart were sampled. Stools were collected from the colon.

### 2.4. The Effect of Treatment with Indole-3-Propionic Acid on Body Weight Gain

14-week-old rats were fed laboratory diet (Labofeed B, Morawski, Poland) ad libitum and received daily intraperitoneal injections of either indole-3-propionic acid (I3P, 30mg/kg b.w. Sigma-Aldrich, St. Luis, MO, USA) [*n* = 9] or a vehicle (20% DMSO Sigma-Aldrich, St. Luis, MO, USA) [*n* = 9] for one week. Body mass was measured every day. Food, water intake, and urine output were measured before and on the last day of the treatment.

### 2.5. Dosage Information

Rats had access to food and water ad libitum. Standard laboratory chow for rodents contains 2 g/kg of tryptophan [23]. In our study, rats received a tryptophan-rich diet (tryptophan content 8.5 g/kg of chow). Consequently, TC and TH rats received daily 141.96 ± 5.96 mg/kg b.w., and 550.56 ± 13.34 mg/kg b.w. of tryptophan, respectively. Dietary recommendations for adult humans suggest 3.5–6 mg/kg b.w. of tryptophan in diet daily [24]. IP3 was administered daily at a dose of 30 mg/kg b.w. to increase IP3 blood concentration approximately 2-fold.

### 2.6. Blood Sampling

Portal vein was catheterized as we described before [19,25]. In short, the portal vein and the superior mesenteric vein were dissected from adjacent tissue and stabilized with ligatures. Subsequently, a catheter was implanted into the portal vein in order to collect blood samples. Afterwards chest cavity was open with longitudinal incision, the heart was exposed, and a needle was inserted into the right ventricle of the heart for systemic blood sampling. Blood was collected for either EDTA tubes for plasma analysis or tubes without anticoagulant for serum analysis.

### 2.7. Stool Sample Collection and Preparation

After taking blood, the rats were killed by decapitation and laparotomy was performed. Intestines were exposed and an approx. 5-cm-long fragment of the colon was ligated and prepared for collection of colon content for metabolic analysis.

A 0.5 mL sample of the colon content (stools) was weighted and homogenized with 1 mL of DMSO in a closed 2 mL laboratory tube by vortexing it for 5 min. Next, the sample was centrifuged for 12 min at 5000 rpm, and 1 mL of the obtained supernatant was transferred to a laboratory tube and again centrifuged for 12 min. All procedures were performed at the temperature of 2–5 °C. The supernatant was collected in Eppendorf tubes and frozen at −20 °C.

### 2.8. Tryptophan and Tryptophan-Derived Metabolites Analysis

Hemolysis-free blood plasma samples and stool supernatant samples were prepared and then were analyzed using UHPLC system SHIMADZU NEXERA (Japan) combined with Photodiode Array Detector SPD-M20A (Shimadzu, Kyoto Japan) and with mass spectrometer LCMS-2020 (Shimadzu, Japan).

To prepare samples for analysis, 100 µL of blood plasma or stool supernatant was treated with 100 µL of MS-grade acetonitrile to deproteinate or to precipitate the mobile phase insoluble impurities. Suspension was vortexed and then filtered through syringe filter (nylon, 0.2 μm). Finally, a four-time dilution of clear solution was made using MS-grade water to obtain solution for analysis. There were two solutions for analysis prepared from each blood plasma sample and stool supernatant sample, and each solution for analysis was analyzed twice.

Solutions for analysis underwent chromatographic separation on Kinetex F5 column (2.6 µm, 100 × 2.1mm, Phenomenex, Torrance, CA, USA) coupled with Security Guard Ultra F5 precolumn (Phenomenex, USA). Injection volume was 5 µL. Binary gradient method with 0.2 ml/min flow rate was set for mobile phase A consist of water and 0.015%(v/v) formic acid and mobile phase B consist of methanol and 0.015%(v/v) formic acid, as follows: 4%B 0min–1.5 min; 4%B–73%B 1.5 min–10.20 min; 73%B 10.20 min–12.20 min. Mass spectrometry was employed as a primary detector for detection and quantification of desired compounds and as a reference detector the Photodiode Array Detector at 280 nm was used to check for any ion suppressions or to verify indole concentration because of indole pure ionization. Mass spectrometer was operating in electrospray ionization mode (ESI) with interface temperature set to 350 °C, desolvation line temperature −250 °C, heat block −200 °C, nebulizing gas −1.5 L/min and drying gas −12 L/min.

Acetonitrile, alcohols and solutions of certain salts are commonly known as denaturizing agents for peptide precipitation. For our purposes, using acetonitrile gave the most acceptable results. Our sample preparation method was developed to easily clear the sample for safe LCMS analysis maintaining the best recovery yield. Similar approach was presented previously [26]. Recovery test was performed using rat plasma with added known amount of standards and yield between 98.0% and 103.0% for desired compounds was achieved (indole = 102.9%; indoxyl = 98.0%; indole-3-acetic acid (I3A) = 98.1%; indole-3-lactic acid (I3L) = 100.8%; indole-3-propionic acid (I3P) = 100.9%; tryptophan (TRP) = 101.5%; serotonin (SER) = 102.7%; kynurenine (KYN) = 98.0%). Considering those recovery yields, all obtained concentration results were treated as satisfying results without recovery compensation.

The levels of indole (IND), [M+H]+ = 118.00; indole-3-carboxylic acid (I3C), [M-H]- = 160.00; tryptamine (TRA), [M+H]+ = 161.00; indole-3-acetic acid (I3A), [M+H]+ =176.00, serotonin (SER), [M+H]+ = 177.00; indole-3-propionic acid (I3P)[M+H]+ = 190.00, indole-3-lactic acid (I3L), [M-H]- = 204.00; tryptophan (TRP), [M+H]+ = 205.00; kynurenine (KYN), [M+H]+ = 209.00; indoxyl sulphate (IS), [M-H]- = 212.00 were evaluated using external standard method (ESTD). The calibration curves were plotted in rages from 0.01 µg/mL to 2.5 µg/mL. Linearity was confirmed for all analyses. All standards were obtained from Sigma-Aldrich and solvents from Merck.

Bacterial metabolite concentration in the stools was calculated as a metabolite concentration in the supernatant multiplied by a factor of 3 (as described above, 1 mL of DMSO was added to 0.5 mL of colon content to prepare supernatant for analysis).

### 2.9. Biochemical Blood and Urine Analyses

Biochemical analysis of hemolysis-free blood samples and urine analyses (creatinine, urea, protein, Na, K) were performed on the Cobas 6000 analyzer (Roche Diagnostics, Indianapolis, IN USA).

### 2.10. ELISA Test

EIAab Kits (Wuhan EIAab Science Co. Ltd., China) were used for the evaluation of vasopressin (cat. no. E1139Ge) and aldosterone (cat. no. E0911Ge) in hemolysis-free blood plasma. All procedures were performed accordingly standard protocol by ELISA Kit Operating Instruction. The absorbance intensity was measured at 450 nm with the Multiskan microplate reader (Termo Fisher Scientific). The tests were performed in duplicate. The obtained results were presented as total protein concentration (pg/mL) for plasma vasopressin and (ng/mL) for aldosterone.

### 2.11. Statistics

The Kolmogorov-Smirnov test was used to test the normality of the distribution. Differences between the groups were evaluated by one-way ANOVA followed by Tukey’s post hoc test (experiments comprising 3 groups) or by *t*-test (experiments comprising 2 groups) if data were normally distributed, otherwise a Kruskal-Wallis test was used. Daily changes in body weight were evaluated by ANOVA for repeated measurements. A value of two-sided *p* < 0.05 was considered significant. Analyses were conducted using Dell Statistica, version 13 (Dell Inc, Tulsa, OK, USA).

## 3. Results

### 3.1. The Effect of Antibiotic Treatment

**Figure 1:**
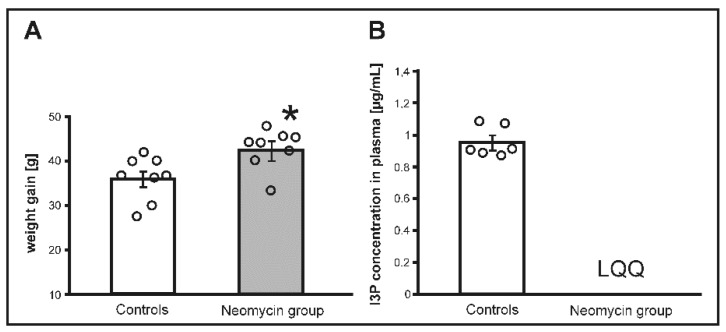
(**A**) Changes in body weight and (**B**) indole-3-propionic (I3P) acid concentration in plasma in Sprague Dawley rats treated with either neomycin, an antibiotic or water (controls) for 14 days. LQQ—below the limit of quantification; * *p* < 0.05 controls vs. neomycin. Means ± SE are presented, circles show individual data points.

There were no significant differences between the groups in body weight before the treatment. Rats receiving antibiotic treatment showed a significantly higher weight gain than untreated rats (*p* < 0.05), (Figure 1). Rats treated with neomycin showed a significantly lower concentration of I3P in portal blood (*p* < 0.0001), whereas the concentration I3P in stools and systemic blood of neomycin treated rats was below the limit of quantification.

There were also significant differences in the concentration of other tryptophan-derived metabolites as presented in Table 2.

### 3.2. The Effect of Tryptophan-Free (TF) and Tryptophan-High (TH) Diets

#### 3.2.1. Weight Gain and Food Intake

**Figure 2:**
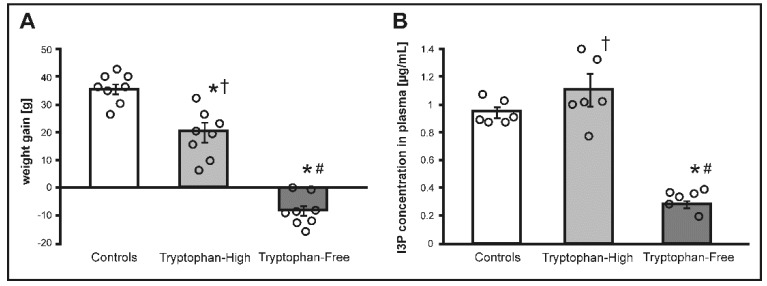
(**A**) Changes in body weight and (**B**) indole-3-propionic acid (I3P) concentration in plasma in Sprague Dawley rats maintained on either standard laboratory chow (control), tryptophan-high chow (TH) or tryptophan-free chow (TF) for two weeks. * *p* < 0.05 vs. control; # *p* < 0.05 vs. TH, † *p* < 0.05 vs. TF. Means ± SE are presented, circles show individual data points.

There were no significant differences between the groups in body weight before the treatment. There was a significant difference between the groups in body weight gain after two weeks of the treatment (F_2.21_ = 72.93, *p* < 0.0001). Specifically, the TF group showed a decrease in body weight. The TH group showed an increase in body weight however, it was a significantly lower than in controls (*p* < 0.001), (Figure 2.).

There was a significant difference in food intake between the groups (F_2.21_ = 38.22, *p* < 0.0001). Specifically, TF rats showed a significantly lower food intake compared to controls (*p* < 0.0001) and TH rats (*p* < 0.0001). The TH group showed also a lower food intake than controls (*p* < 0.01). TF rats showed a moderately lower caloric intake than controls and the TH group; however, ANOVA revealed no significant difference in caloric intake between the groups (Table 3).

#### 3.2.2. Serum Protein and Water-Electrolyte Balance

There were significant differences between the groups in total serum protein level (F_2.15_ = 7.12, *p* < 0.01). Specifically, the TH group had a significantly higher (*p* < 0.01) total serum protein compared to the TF group. There was also a trend towards lower total serum protein in TF rats compared to controls (*p* = 0.06). There were no significant differences in water intake between the groups. TF rats showed a higher urine output than the TH group and controls; however, ANOVA revealed only a trend for differences between the groups in urine output (F_2.21_ = 5.72, *p* = 0.06). There was a significant difference in urine specific gravity between the groups (F_2.21_ = 5.3, *p* < 0.01). Specifically, TF rats had a significantly lower urine-specific gravity compared to TH rats (*p* < 0.01) and a trend towards lower urine-specific gravity compared to controls (*p* = 0.06). There was no significant difference in serum sodium and potassium between the groups. There was a significant difference in serum urea between the groups (F_2.15_ = 20.76, *p* < 0.0001). Namely, TH rats had a significantly higher serum concentration of urea compared to controls (*p* < 0.001) and TF rats (*p* < 0.001). The TF group had a lower plasma vasopressin concentration; however, ANOVA showed only a trend for differences between the three experimental groups (F_2.15_ = 1.91, *p* = 0.18, Table 4).

#### 3.2.3. Concentrations of Tryptophan and Tryptophan Metabolites

In general, in comparison to controls, the TF group had lower, whereas the TH group had higher stool and blood concentrations of tryptophan, tryptophan-derived endogenously synthetized mediators and bacteria-produced tryptophan derivates (Table 3). Specifically, there was a significant difference in tryptophan concentration in stools between the groups (F_2.15_ = 10.57, *p* < 0.001), i.e., TH rats had a significantly higher tryptophan concentration compared to TF rats (*p* < 0.001) and controls (*p* < 0.01). Similarly, there were significant differences in portal blood concentration of tryptophan between the groups (F_2.15_ = 17.65, *p* < 0.0001), i.e., the TF group had a significantly lower tryptophan concentration in portal and systemic blood in comparison to controls and the TH group, (Table 3). This was accompanied by a significantly higher blood concentration of endogenously synthetized tryptophan-derived hormones such as serotonin and kynurenine in the TH group (Table 3).

There were significant differences in IP3 concentration between the groups in stools (F_2.15_ = 22.61, *p* < 0.0001), portal blood (F_2.15_ = 39.98, *p* < 0.0001) and systemic blood (F_2.15_ = 33.11, *p* < 0.0001). The TH group had a significantly higher I3P concentration in stools compared to controls (*p* < 0.01) and TF rats (*p* < 0.0001). I3P concentration in stools in TF rats was a significantly lower compared to controls (*p* < 0.01). TH rats had a significantly higher I3P concentration in portal blood compared to controls (*p* < 0.001) and the TF group (*p* < 0.0001). I3P concentration in portal blood of TF rats was significantly lower compared to controls (*p* < 0.01). TF rats also had a significantly lower I3P concentration in systemic blood compared to controls (*p* < 0.001), and the TH group (*p* < 0.0001).

### 3.3. The Effect of Treatment with I3P on Body Weight Gain

**Figure 3:**
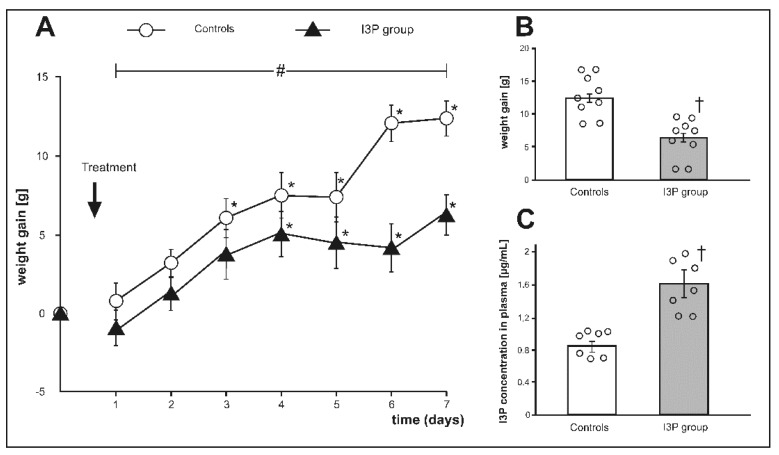
(**A**) Changes in body weight during the experiment, (**B**) weight gain at the end of experiment, and (**C**) plasma indole-3-propionic acid (I3P) concentration at the end of experiment in Sprague Dawley rats treated with either indole-3-propionic acid (I3P group) or the vehicle (controls). * *p* < 0.05 vs. baseline, # *p* < 0.05 (time × group interaction) by ANOVA for repeated measurements, † *p* < 0.05 vehicle vs. I3P group. Means ± SE are presented, circles show individual data points.

Before the treatment, there was no significant difference between the groups in body weight (g) 324.52 ± 9.58 and 328.04 ± 9.80, controls and the I3P group, respectively. There was a significant difference in body weight gain during the treatment between the groups (F_6.96_ = 4.156, *p* < 0.001, ANOVA, time × group interaction, Figure 3A). At the end of the experiment, rats treated with I3P showed a significantly lower weight gain than controls i.e., 6.34 ± 1.30. vs. 12.53 ± 1.12 (Figure 3B), which was associated with approximately 2-fold higher plasma concentration of I3P in the I3P treated group (Figure 3C).

There was no significant difference between the two groups with respect to water-electrolyte balance (Table 5).

## 4. Discussion

A new finding of our study is that I3P, a tryptophan-derived gut bacteria metabolite, contributes to changes in body weight gain produced by antibiotics and tryptophan-rich diet.

Recent evidence suggests that gut bacteria and their metabolites affect many biological processes [14,16,18]. Here, we found that treatment with neomycin, a poorly absorbable antibiotic used for intestinal decontamination (e.g., in liver failure patients), increased weight gain in rats. Our findings are in line with previous studies showing that antibiotic treatment increases body weight in mammals [20] and poultry [21]. However, the mechanisms of a positive effect of antibiotics on weight gain remain obscure.

In further experiments, we found that tryptophan-rich diet reduced body weight gain in rats. A decrease in body weight gain due to increased tryptophan intake has also previously been reported by others [10,11,21]. However, again, the mechanisms have not been elucidated.

In our study, changes in body weight gain due to treatment with the antibiotic and due to tryptophan-rich diet were associated with significant changes in the concentration of tryptophan-derived bacterial metabolites in stools and in blood. Therefore, we hypothesized that an association between changes in gut bacteria metabolites of tryptophan and changes in body weight gain may be of a causative nature. Here, we found that changes in body weight gain matched the most changes in the concentration of I3P. For this reason, in a separate series of experiments we evaluated the effect of I3P treatment on body weight gain in rats. In fact, rats treated with I3P showed a 2-fold lower weight gain than rats treated with the vehicle. This suggests that I3P may be a link between tryptophan-rich diet and lower body weight gain.

Several biological effect of I3P have been reported previously. Specifically, I3P has been suggested to be a free-radical scavenger [18] and to reduce the oxidative stress associated with formation of β-amyloid in Alzheimers’s disease [27].

Finally, we found that tryptophan-depleted diet negatively affects body weight gain, which is in line with previous studies [7,8,9]. Rats on tryptophan-free diet had a lower concentration of I3P in stool and blood, which should have a positive effect on body weight gain in this group of rats. However, it seems that the potentially positive effect of decreased I3P level on body weight gain was surpassed by the negative effect of deficiency of tryptophan, an essential amino acid. Our findings imply at least two mechanisms contributing to weight loss in tryptophan depleted rats, i.e., a decrease in anabolism reflected in lower serum protein, and an increase in urinary excretion. The latter could be caused by increased glomerular filtration rate (decreased serum protein) and decreased concentration of urine by the kidney (decreased urea and vasopressin levels) in tryptophan-depleted rats.

Interestingly, despite two weeks of treatment with tryptophan-free diet the TF group showed the presence of tryptophan in stools and blood (approx. 2-fold lower concentrations than controls). Whereas tryptophan in the blood may originate from catabolism of body proteins, the presence of tryptophan in stool suggests its bacterial origin. In this regard, it has been shown that several bacteria, including *Escherichia coli* and *Corynebacterium* may produce tryptophan [28], which requires phosphoenolpyruvate and erythrose 4-phosphate that originate from many biochemical reactions [29].

A limitation of our study is that we tested only one group of bacterial products, i.e., tryptophan-derived indoles. It is likely that both the antibiotic and tryptophan-rich diet affected production of other bacterial metabolites such as trimethylamines, short-chain fatty acids, hydrogen sulfide or other [30,31,32,33], which exert biological effects. Arguably, the rapid development of ultra-high-performance liquid chromatography with mass spectrometry [26,34,35] will make it possible in the future to identify many other bacterial products with biological activity. More studies evaluating the effect of an interaction between tryptophan and gut bacteria on mammals’ energy balance are needed. For example, evaluation of gut bacteria composition in rats treated with antibiotic and tryptophan could bring additional insights to mechanisms of our findings.

## 5. Conclusions

In summary, a new finding of our study is that bacterial metabolites of tryptophan such as I3P may contribute to changes in body weight gain produced by antibiotic treatment and tryptophan-rich diet. Further studies are needed to clarify the effect of bacterial metabolites on body weight in mammals.

## Figures and Tables

**Table 1:** Energy, tryptophan and other nutrient content in the laboratory chows used in the study.

Diet Composition	TC	TH	TF
Tryptophan (g/kg)	2	8.5	0
Metabolizable Energy (MJ)	11.5	13.5	16.5
Crude protein (%)	17.4	19.1	16.4
Crude fat (%)	3.5	3.3	7
Crude fiber (%)	7	4.9	5
Crude ash (%)	3.2	6.4	3.5
Starch (%)	33	36.5	28.9

Standard laboratory chow (TC), tryptophan-high chow (TH) and a tryptophan-free chow (TF).

**Table 2:** Metabolic parameters and concentrations of tryptophan and its metabolites in stools, portal blood and systemic blood in Sprague Dawley rats maintained on standard laboratory diet and treated with either a water solution of neomycin, an antibiotic (neomycin group) or tap water (controls) for two weeks.

Group	Controls	Neomycin Group
**Metabolic Parameters**		
Body mass at the beginning of the experiment (g)	279.37 ± 15.81	276.07 ± 16.16
Weight gain (g)	36.16 ± 2.17	42.56 ± 3.99 *
Food intake (g)	22.40 ± 0.61	22.16 ± 0.72
Caloric intake (kcal)	61.51 ± 1.56	61.24 ± 1.71
Water intake (mL)	33.97 ± 1.61	34.80 ± 1.05
Urine output (mL)	17.29 ± 1.23	13.20 ± 1.29 *
Stool output (g)	9.10 ± 1.00	13.30 ± 0.78 *
**Tryptophan and Tryptophan Metabolites Concentration**		
**Tryptophan**		
Diet (intake mg/24 h)	44.80 ± 1.23	44.60 ± 1.44
Diet (intake mg/kg b.w./24 h)	143.57 ± 6.17	141.14 ± 6.00
Stools (µg/mL)	2.15 ± 0.47	2.22 ± 0.33
Portal blood (µg/mL)	10.74 ± 1.08	9.04 ± 0.97
Systemic blood (µg/mL)	8.09 ± 0.46	6.73 ± 0.47
**Kynurenine**		
Portal blood (µg/mL)	0.36 ± 0.04	0.41 ± 0.03
Systemic blood (µg/mL)	0.48 ± 0.03	0.38 ± 0.01 *
**Serotonin**		
Portal blood (µg/mL)	0.74 ± 0.10	0.58 ± 0.05
Systemic blood (µg/mL)	0.69 ± 0.02	0.63 ± 0.04
**Indole**		
Stools (µg/mL)	63.61 ± 18.74	6.63 ± 1.60 *
**Indoxyl Sulfate**		
Portal blood (µg/mL)	4.76 ± 0.47	1.96 ± 0.44 *
Systemic blood (µg/mL)	4.80 ± 0.31	3.00 ± 0.44 *
**Indole-3-Acetic Acid**		
Stools (µg/mL)	1.51 ± 0.27	0.96 ± 0.09
Portal blood (µg/mL)	0.14 ± 0.02	0.23 ± 0.07
Systemic blood (µg/mL)	0.08 ± 0.01	0.11 ± 0.03
**Indole-3-Propionic Acid**		
Stools (µg/mL)	1.89 ± 0.37	LQQ
Portal blood (µg/mL)	1.10 ± 0.09	0.08 ± 0.02 *
Systemic blood (µg/mL)	0.96 ± 0.04	LQQ
**Indole-3-Lactic Acid**		
Stools (µg/mL)	0.88 ± 0.41	2.34 ± 0.51 *
Portal blood (µg/mL)	0.09 ± 0.01	0.10 ± 0.01
Systemic blood (µg/mL)	0.07 ± 0.01	0.10 ± 0.01
**Indole-3-Carboxylic Acid**		
Stools (µg/mL)	LQQ	LQQ

LQQ—below the limit of quantification; * *p* < 0.05 for comparison between the groups. Means ± SE are presented.

**Table 3:** Weight gain, metabolic parameters and concentrations of tryptophan and its metabolites in stools, portal blood and systemic blood in Sprague Dawley rats maintained on either standard laboratory chow (TC), tryptophan-high chow (TH) or tryptophan-free chow (TF) for two weeks.

Group	TC	TH	TF
**Metabolic Parameters**			
Body mass at the beginning of the experiment (g)	288.95 ± 16.71	288.91 ± 10.01	278.43 ± 11.03
Weight gain (g)	36.11 ± 1.88	20.30 ± 3.69 *^,†^	−8.26 ± 1.83 *^,#^
Food intake (g)	22.80 ± 0.66	19.98 ± 0.59 *^,†^	14.57 ± 0.76 *^,#^
Caloric intake (kcal)	62.60 ± 1.71	64.40 ± 1.91	57.44 ± 2.80
Water intake (mL)	33.18 ± 1.61	33.55 ± 1.33	32.07 ± 3.87
Urine output (mL)	17.13 ± 0.99	16.50 ± 0.98	23.25 ± 3.36
Stool output (g)	9.38 ± 0.91	12.39 ± 0.81 *^,†^	2.10 ± 0.15 *^,#^
**Tryptophan and Tryptophan Metabolites Concentration**			
**Tryptophan**			
Diet (intake mg/24 h)	45.60 ± 1.33	169.79 ± 5.04 *	0
Diet (intake mg/kg b.w./24 h)	141.96 ± 5.96	550.56 ± 13.34	0
Stools (µg/mL)	2.36 ± 0.45	4.39 ± 0.48 *^,†^	1.66 ± 0.37 ^#^
Portal blood (µg/mL)	10.65 ± 1.08	13.60 ± 1.06 ^†^	6.58 ± 0.47 *^,#^
Systemic blood (µg/mL)	8.18 ± 0.47	9.11 ± 0.27 ^†^	5.60 ± 0.43 *^,#^
**Kynurenine**			
Portal blood (µg/mL)	0.37 ± 0.04	0.73 ± 0.08 *^,†^	0.13 ± 0.03 *^,#^
Systemic blood (µg/mL)	0.47 ± 0.03	0.59 ± 0.09 ^†^	0.20 ± 0.02 *^,#^
**Serotonin**			
Portal blood (µg/mL)	0.72 ± 0.10	0.86 ± 0.10 ^†^	LQQ
Systemic blood (µg/mL)	0.68 ± 0.03	0.82 ± 0.07 ^†^	0.64 ± 0.02 ^#^
**Indole**			
Stools (µg/mL)	22.99 ± 15.92	59.13 ± 18.07	12.46 ± 1.72
**Indoxyl sulfate**			
Portal blood (µg/mL)	4.73 ± 0.47	3.08 ± 0.51 *	1.75 ± 0.17 *
Systemic blood (µg/mL)	4.77 ± 0.31	2.95 ± 0.18 *^,†^	1.79 ± 0.13 *^,#^
**Indole-3-Acetic Acid**			
Stools (µg/mL)	1.46 ± 0.28	0.65 ± 0.17 *	0.31 ± 0.07 *
Portal blood (µg/mL)	0.15 ± 0.02	0.17 ± 0.07	0.04 ± 0.01
Systemic blood (µg/mL)	0.08 ± 0.01	0.05 ± 0.01 *^,†^	0.023 ± 0.004 *^,#^
**Indole-3-Propionic Acid**			
Stools (µg/mL)	2.13 ± 0.34	3.74 ± 0.47 *^,†^	0.54 ± 0.09 *^,#^
Portal blood (µg/mL)	1.11 ± 0.08	2.04± 0.22 *^,†^	0.28 ± 0.01 *^,#^
Systemic blood (µg/mL)	0.95 ± 0.04	1.12 ± 0.12 ^†^	0.29 ± 0.03 *^,#^
**Indole-3-Lactic Acid**			
Stools (µg/mL)	0.79 ± 0.40	0.61 ± 0.21	0.31 ± 0.06
Portal blood (µg/mL)	0.09 ± 0.02	0.24 ± 0.09 ^†^	0.04 ± 0.01 ^#^
Systemic blood (µg/mL)	0.07 ± 0.01	0.14 ± 0.02 *^,†^	0.039 ± 0.003 ^#^
**Indole-3-Carboxylic Acid**			
Stools (µg/mL)	LQQ	0.28 ± 0.03 *^,†^	LQQ

* *p* < 0.05 vs. TC, # *p* < 0.05 vs. TH, † *p* < 0.05 vs. TF. Means ± SE are presented.

**Table 4:** Electrolyte balance, aldosterone and vasopressin blood level in Sprague Dawley rats maintained on either standard laboratory chow (TC), tryptophan-high chow (TH) or tryptophan-free chow (TF) for twee weeks.

Group	TC	TH	TF
**Electrolyte Intake with Food**			
Sodium (mmol/24 h)	1.88 ± 0.05	2.09 ± 0.06 ^†^	1.33 ± 0.07 *^,#^
Potassium (mmol/24 h)	4.37 ± 0.12	4.70 ± 0.13 ^†^	1.94 ± 0.09 *^,#^
**Serum total Protein and Electrolytes**			
Total Protein (g/dL)	5.28 ± 0.07	5.45 ± 0.03 ^†^	4.95 ± 0.15 ^#^
Sodium (mmol/L)	140.17 ± 1.30	141.00 ± 0.45	138.83 ± 1.62
Potassium (mmol/L)	4.82 ± 0.30	4.41 ± 0.14	4.40 ± 0.22
Creatinine (mg/dL)	0.78 ± 0.08	0.68 ± 0.03	0.72 ± 0.07
Urea (mg/dL)	54.17 ± 2.32	73.5 ± 1.52 *^,†^	50.67± 3.77 ^#^
**Urinalysis**			
Specific gravity (g/L)	1.036 ± 0.002	1.039 ± 0.002 ^†^	1.026 ± 0.004 ^#^
Creatinine (mg/dL)	61.69 ± 5.71	64.11 ± 3.77 ^†^	40.82 ± 6.21 *^,#^
Sodium (mmol/L)	64.50 ± 6.66	110.63 ± 9.13 *^,†^	62.75 ± 12.67 ^#^
Sodium excretion (mmol/24 h)	1.08 ± 0.09	1.78 ± 0.10 *^,†^	1.23 ± 0.14 ^#^
Potassium (mmol/L)	247.58 ± 18.79	260.44 ± 16.33 ^†^	98.26 ± 17.30 *^,#^
Potassium excretion (mmol/24 h)	4.14 ± 0.20	4.20 ± 0.12 ^†^	2.28 ± 0.16 *^,#^
**Plasma Vasopressin an Aldosterone**			
Vasopressin (pg/mL)	1500.2 ± 140.9	1304.6 ± 125.1	1171.1 ± 86.5
Aldosterone (ng/mL)	9.627 ± 1.620	8.706 ± 0.472	8.392 ± 0.649

* *p* < 0.05 vs. TC, ^#^ *p* < 0.05 vs. TH, † *p* < 0.05 vs. TF (by post-hoc Tukey’s test preceded by ANOVA). Means ± SE are presented.

**Table 5:** Metabolic and water-electrolyte parameters at the beginning and at the end of experiments in rats treated with indole-3-propionic acid (I3P group) and rats treated with the vehicle (controls).

Group	Controls		I3P	
	Before the treatment	At the end of treatment	Before the treatment	At the end of treatment
Food intake (g)	20.79 ± 0.49	18.77 ± 0.55 *	21.11 ± 0.50	19.89 ± 0.71
Caloric intake (kcal)	57.08 ± 1.36	51.55 ± 1.50 *	57.98 ± 1.37	54.61 ± 1.96
Water intake (mL)	28.86 ± 1.57	27.71 ± 2.17	29.26 ± 2.14	27.57 ± 2.77
Urine output (mL)	14.43 ± 1.21	15.86 ± 1.16	13.43 ± 1.07	14.29 ± 1.13
Stool output (g)	8.9 ± 0.72	8.01 ± 0.70	8.56 ± 0.52	7.43 ± 0.60
**Serum Electrolytes and Total Protein**				
Sodium (mmol/L)		143.14 ± 0.88		142.71 ± 1.06
Potassium (mmol/L)		4.47 ± 0.11		4.54 ± 0.05
Creatinine (mg/dL)		0.50 ± 0.02		0.54 ± 0.06
Urea (mg/dL)		51.57 ± 2.35		53.71 ± 3.56
Total Protein (g/dL)		5.30 ± 0.03		5.24 ± 0.07
**Urinalysis**				
Specific gravity (g/L)	1.039 ± 0.001	1.035 ± 0.001 *	1.043 ± 0.003	1.038 ± 0.001
Daily sodium excretion (mmol)	1.07 ± 0.08	1.01 ± 0.04	0.89 ± 0.08	0.91 ± 0.04
Daily potassium excretion (mmol)	3.25 ± 0.17	3.11 ± 0.12	3.38 ± 0.09	3.03 ± 0.08 ^#^

* *p* < 0.05 vs. before the treatment in controls, # *p* < 0.05 vs. before the treatment in the I3P group. Means ± SE are presented.
